# ﻿*Gracilariopsisgrevogerungii* (Gracilariales, Rhodophyta), a new species of marine algae from Indonesia

**DOI:** 10.3897/phytokeys.259.154294

**Published:** 2025-07-10

**Authors:** Ga Hun Boo, Il Ki Hwang

**Affiliations:** 1 Department of Marine Biology, Pukyong National University, Busan 48513, Republic of Korea Pukyong National University Busan Republic of Korea; 2 Seaweed Research Institute, National Institute of Fisheries Science, Haenam 59002, Republic of Korea Seaweed Research Institute, National Institute of Fisheries Science Haenam Republic of Korea

**Keywords:** Agar-yielding, Gracilariaceae, molecular marker, morphology, Southeast Asia

## Abstract

*Gracilariopsisgrevogerungii* G.H.Boo & I.K.Hwang, **sp. nov.**, a new species from southern Indonesia, is described based on morphological features and molecular data from two genes: the plastid-encoded *rbc*L and the mitochondrial COI-5P. *Gracilariopsisgrevogerungii* can be distinguished from other species in the same genus by a combination of traits: terete main axes with irregular branches and scarce short branchlets, and hemispherical cystocarps with up to 3 ostioles and 9–14 cell-layered pericarp. The species occurred on sandy-muddy substrates in the intertidal to the shallow subtidal zone. Phylogenies based on *rbc*L and COI-5P revealed its sister relationship with the subclade of *Gp.heteroclada* from China and *Gp.mclachlanii* from Tanzania. Our results highlight the need for further findings of the agar-yielding Gracilariaceae in southern Indonesia, expanding our knowledge of red algal diversity in tropical Southeast Asia.

## ﻿Introduction

*Gracilariopsis* E.Y.Dawson (Gracilariaceae) is generally known as one of the major sources of agar used in food, healthcare and biotechnology industries. *Gracilariopsis* was established by Dawson (1949) to accommodate species that were previously placed in *Gracilaria* Greville and presented a small–celled, broad-based gonimoblast and the absence of nutritive filaments connecting gonimoblast with the pericarp. The established type species for the genus was *Gp.sjoestedtii* (Kylin) E.Y.Dawson. However, Papenfuss (1967) merged *Gracilariopsis* into *Gracilaria* because of the lack of morphological difference at the genus level based on observations of *Gp.sjoestedtii* and *Gracilariaverrucosa* (Hudson) Papenfuss, the generitype of *Gracilaria*. Ohmi (1958) reinstated *Gracilariopsis* based on the observation of *Gp.chorda* (Holmes) Ohmi from Japan, whereas Yamamoto (1978) included it within *Gracilaria*, despite his beautiful illustrations showing the superficial formation of spermatangia and the absence of nutritive tubular filaments in cystocarps. Fredericq and Hommersand (1989) reinstated *Gracilariopsis* including four species which have superficial spermatangia and lack nutritive tubular cells. *Gracilariopsislemaneiformis* (Bory) Dawson, Acleto & Foldvik was designated as the generitype because of its priority over *Gp.sjoestedtii*.

*Gracilariopsis* has been robustly supported in phylogenies based on nuclear small subunit ribosomal DNA (SSU rDNA) and plastid *rbc*L gene sequence (Bird et al. 1994; Gurgel et al. 2003a, b). Gurgel et al. (2003b) designated the generitype of *Gracilariopsis* as *Gp.andersonii* (Grunow) Dawson, based on material from the northwest coast of America. The authors confirmed that *Gp.lemaneiformis*, for a long time considered a widespread species, is likely restricted to the Peruvian coast, and the collections of *Gp.lemaneiformis* from northwestern America correspond to *Gp.andersonii*. They also indicated that the collections of *Gp.lemaneiformis* from China and Japan may represent an undescribed species that is related to *Gp.heteroclada* J.-F.Zhang & B.-M.Xia.

The knowledge on species diversity of *Gracilariopsis* has improved with the increase of sequences availability, especially COI-5P and *rbc*L data from various regions (Bellorin et al. 2008; Gurgel et al. 2003a, b; Iyer et al. 2005; Le and Lin 2006; Lin 2008; Muangmai et al. 2014; Suzuki and Terada 2022). Recently, phylogenetic relationships of species within *Gracilariopsis* were further investigated using organellar genomes (Iha et al. 2018; Lyra et al. 2021). To date, a total of 23 species have been listed in the AlgaeBase (Guiry and Guiry 2025). Because most species of *Gracilariopsis* lack distinctive vegetative and reproductive characteristics that allow for reliable differentiation from other species (Bellorin et al. 2008), DNA sequence data is needed to evaluate the species diversity of *Gracilariopsis*.

Weber-van Bosse (1928) reported *Gracilariopsislemaneiformis* as *Gracilarialemaneiformis* (Bory) Greville in Indonesia, but it has not been collected since (Meinita et al. 2021). The objectives of this study were to assess the occurrence of *Gracilariopsis* species in southern Indonesia and to elucidate the taxonomic identities of the species present. During collection trips in southern Indonesia, several *Gracilariopsis*-like plants were collected from Nusa Lembongan Island, Bali. Based on detailed morphological comparisons and analyses of plastid *rbc*L and mitochondrial COI-5P sequences, we recognize these specimens as representing a new species of *Gracilariopsis*.

## ﻿Materials and methods

Specimens were collected in June 2017 at Tamarind Beach (8°40'47.51"S, 115°26'10.20"E), Nusa Lembongan Island, Bali, Indonesia (Table 1). Specimens were mounted on herbarium sheets and tissue samples were dehydrated in silica gels for DNA sequencing. For anatomical observation, plants were sectioned using razor blades and were stained with 1% aqueous aniline blue. Photographs were taken with a DP-71 camera (Olympus, Tokyo, Japan) mounted on a BX-51 microscope (Olympus). Vouch specimens are housed at the Herbarium of the Department of Marine Biology, Pukyong National University, Busan, Korea (**PKNU**).

**Table 1.** Information of collection and GenBank accession number of *Gracilariopsisgrevogerungii* used in the present study.

Voucher code	Collection site and date	COI-5P	*rbc*L
PKNU00672 (isotype)	Tamarind Beach, Nusa Lembongan Island, Bali, Indonesia; 8°40'47.51"S, 115°26'10.20"E; 21.vi.2017	PV106179	PV106181
PKNU00673 (holotype)	Tamarind Beach, Nusa Lembongan Island, Bali, Indonesia; 8°40'47.51"S, 115°26'10.20"E; 21.vi.2017	PV106180	PV106182
PKNU00674 (isotype)	Tamarind Beach, Nusa Lembongan Island, Bali, Indonesia; 8°40'47.51"S, 115°26'10.20"E; 21.vi.2017	PV424435	–
PKNU00676 (isotype)	Tamarind Beach, Nusa Lembongan Island, Bali, Indonesia; 8°40'47.51"S, 115°26'10.20"E; 21.vi.2017	PV424436	–

DNA extraction, polymerase chain reaction amplification, and sequencing procedures followed Boo et al. (2016). The primer set used for amplifying and sequencing was F145, F754, R898, and R1442 for plastid *rbc*L (Kim et al. 2010), and GazF1 and GazR1 for mitochondrial COI-5P (Saunders 2005). All sequences were aligned together with publicly available sequences of *Gracilariopsis* species in GenBank, using the MUSCLE algorithm in MEGA7 (Kumar et al. 2016) with default parameters and the alignment was manually adjusted. *Curdiearacovitzae* Hariot, *Gracilariavermiculophylla* (Ohmi) Papenfuss, and *Melanthaliaobtusata* (Labillardiere) J.Agardh were used as outgroups based on previous studies of the Gracilariaceae (Gurgel and Fredericq 2004; Bellorin et al. 2008; Iha et al. 2018). Sequences generated in the present study were deposited in GenBank (PV106179–PV106182, PV424435, PV424436).

Phylogenies of both datasets were reconstructed using maximum likelihood (ML) and Bayesian inference (BI). The ML analysis was performed using the W-IQ-tree webserver (Trifinopoulos et al. 2016) with 1,000 ultrafast bootstrap (BS) replications (-bb 1000) and model test option (-m TEST). The BI analysis was performed with MrBayes v.3.2.1 (Ronquist et al. 2012) using the Metropolis-coupled Markov Chain Monte Carlo (MC3) with the best-fitting substitution model selected by IQ-tree. Four million generations of two independent runs were performed with four chains and sampling trees every 100 generations. The burn-in period was identified graphically by tracking the likelihoods at each generation to determine whether they reached a plateau. Twenty-five percent of saved trees were removed, and the remaining trees were used to infer Bayesian posterior probabilities (BPP).

## ﻿Results

### ﻿Molecular phylogeny

**Figure 1.**
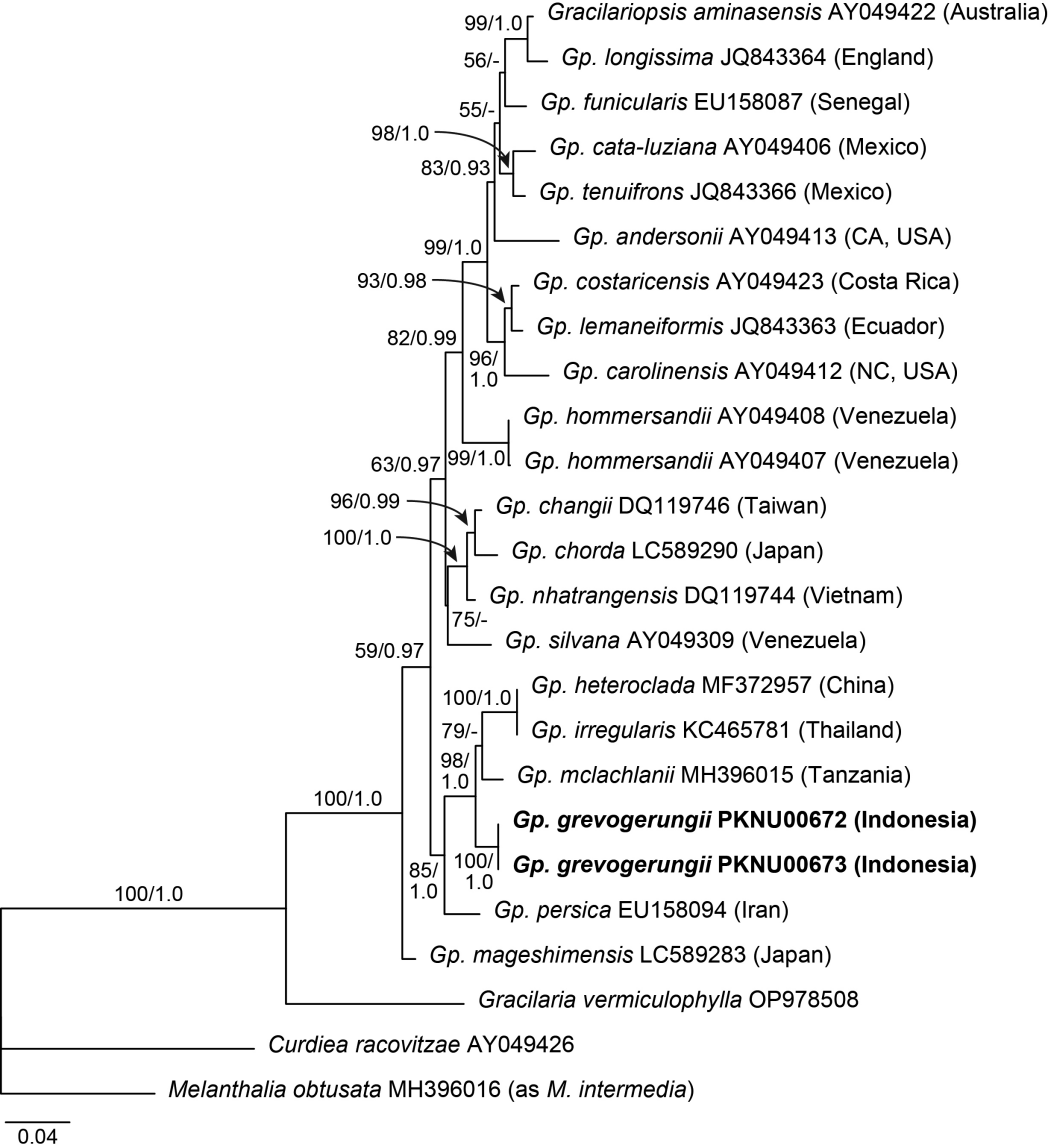
Maximum likelihood (ML) phylogeny of *Gracilariopsis* using plastid *rbc*L sequences. ML bootstrap values (≥50%) and Bayesian posterior probabilities (≥0.9) are shown at branches. Bold letters indicate *Gp.grevogerungii* sp. nov.

Six sequences were generated in the present study, two *rbc*L and four COI-5P sequences. A total of 25 *rbc*L sequences were aligned, including 23 publicly available sequences of *Gracilariopsis* and three outgroups. In the *rbc*L phylogeny (Fig. 1), the Indonesian taxon was distinct from the other species in the genus, and formed a sister relationship with the clade of *Gp.heteroclada*, *Gp.irregularis* (I.A.Abbott) N.Muangmai, A.Chirapart & A.Lewmanomont, and *Gp.mclachlanii* Buriyo, Bellorin & M.C.Oliveira (98% MLBS, 1.0 BPP). The pairwise divergence of *rbc*L sequences between the Indonesian taxon and related species was 2.6–3.3%, with identical sequences among the Indonesian specimens.

**Figure 2.**
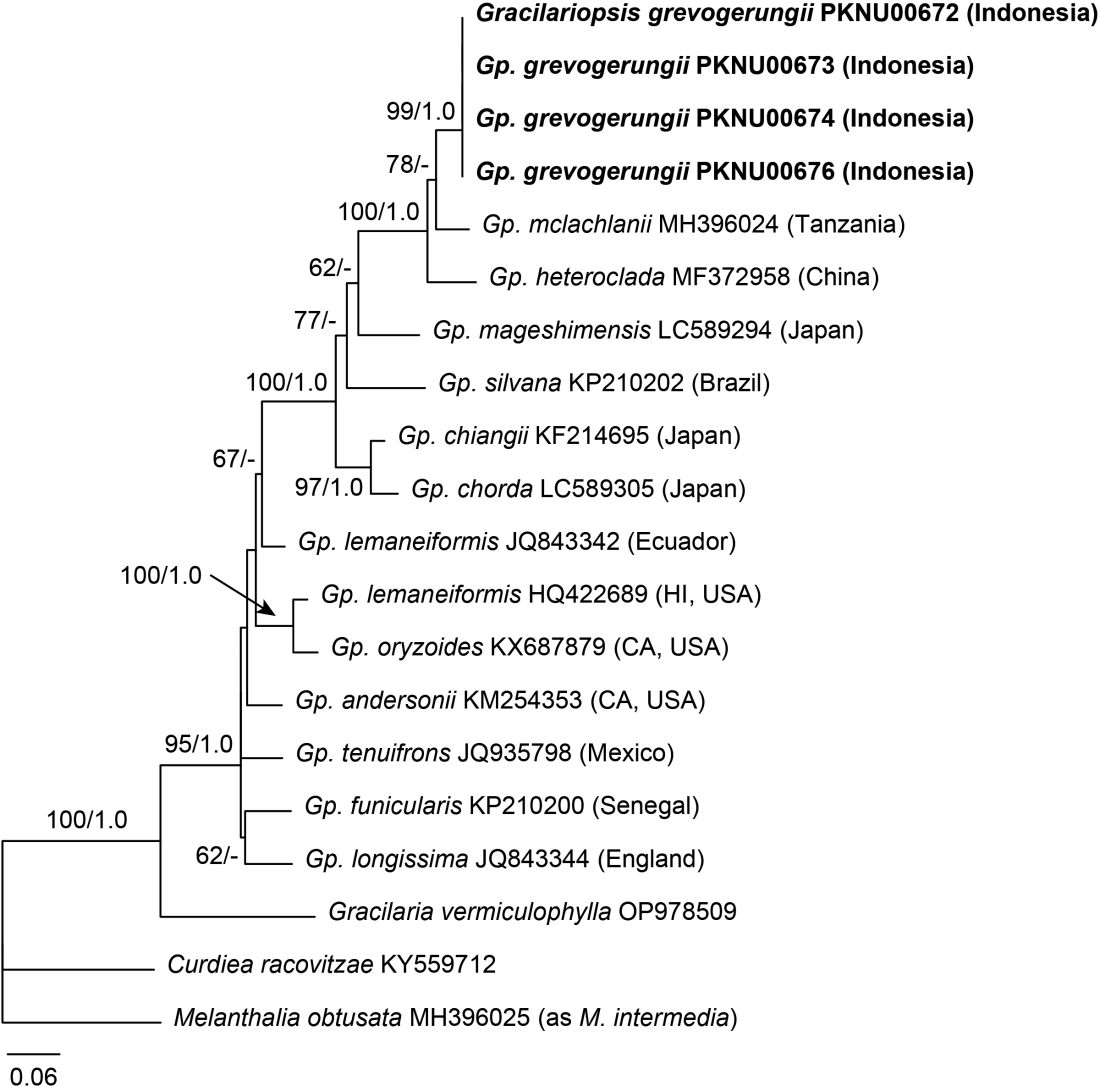
Maximum likelihood (ML) phylogeny of *Gracilariopsis* using mitochondrial COI-5P sequences. ML bootstrap values (≥50%) and Bayesian posterior probabilities (≥0.9) are shown at branches. Bold letters indicate *Gp.grevogerungii* sp. nov.

In the COI-5P phylogeny (Fig. 2), the Indonesian taxon placed in a position largely congruent with that in the *rbc*L phylogeny, forming a clade with *Gp.heteroclada* and *Gp.mclachlanii* (100% MLBS, 1.0 BPP). The pairwise divergence of COI-5P sequences between the Indonesian taxon and related species was 4.7–6.1%, with identical sequences among the Indonesian specimens.

### ﻿Morphological observations

**Figure 3.**
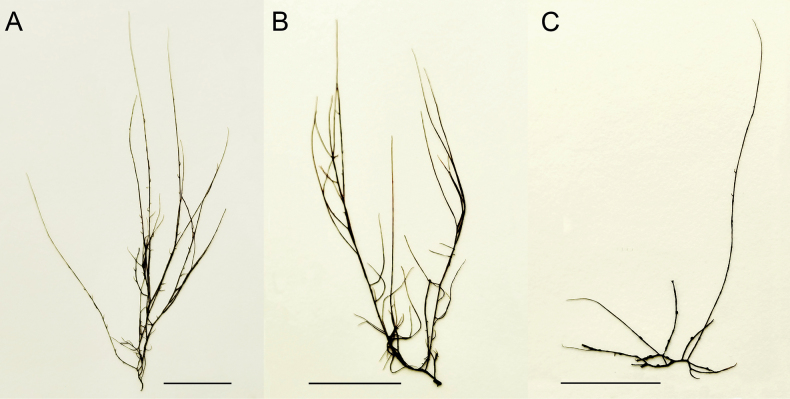
**A–C.** Habit of *Gracilariopsisgrevogerungii* sp. nov. **A.** Holotype specimen (PKNU00673) from Tamarind Beach, Nusa Lembongan Island, Bali, Indonesia; 21 June, 2017; **B.** Isotype specimen having tetrasporangia (PKNU00676); **C.** Isotype specimen bearing cystocarps (PKNU00672). Scale bars: 2 cm (**A–C**).

**Figure 4.**
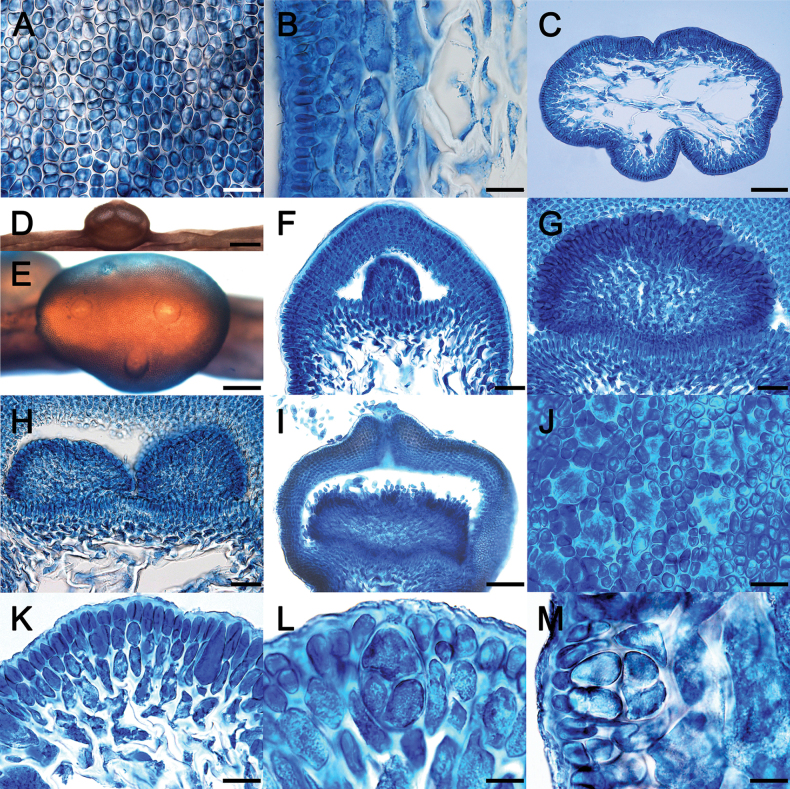
**A–M.** Morphology and anatomy of *Gracilariopsisgrevogerungii* sp. nov. **A.** Surface view of axis showing irregularly arranged cortical cells; **B.** Longitudinal section of axis showing compact cortex and medulla; **C.** Transverse section of axis showing abrupt transition in cells size from cortex to medulla; **D.** Hemispherical cystocarp; **E.** Three ostioles on a single cystocarp; **F.** Longitudinal section of cystocarp showing early stage of gonimoblast; **G.** Longitudinal section of cystocarp lacking nutritive tubular cells between gonimoblast and pericarp; **H.** Two gonimoblasts sometimes formed within a single cystocarp; **I.** Longitudinal section of cystocarp showing releasing carpospores through ostiole; **J.** Tetrasporangia partially immersed by cortical cells; **K.** Tetrasporangial initial formed from inner cortical cells; **L, M.** Decussately to cruciately divided tetrasporangia. Scale bars: 20 μm (**A, B, J–M**); 100 μm (**C, I**); 400 μm (**D**); 200 μm (**E**); 40 μm (**F–H**).

Details of morphological features are introduced in description and illustration below (Figs 3, 4). The *Gracilariopsis* species from Indonesia is characteristic of the genus in the absence of nutritive tubular cells between the gonimoblast and pericarp. The species can be distinguished from other species of *Gracilariopsis* by a combination of terete main axes with irregular branches with sparse, filiform branchlets (up to 4 mm), scattered tetrasporangia on axes and branches, and hemispherical cystocarps with up to 3 ostioles and 9–14 cell-layered pericarps. A morphological comparison of Indonesian *Gracilariopsis* with other terete species of the genus is given in Table 2. However, without DNA sequences, it is difficult to recognize the Indonesian *Gracilariopsis*.

**Table 2.** Morphology and distribution of *Gracilariopsisgrevogerungii* and similar species.

	*Gp.grevogerungii* G.H.Boo & I.K.Hwang, sp. nov.	*Gp.chiangii* Showe M.Lin 2008	*Gp.chorda* (Holmes) Ohmi 1958	*Gp.heteroclada* J.-F.Zhang & B.-M.Xia 1991	*Gp.irregularis* (Abbott) Muangmai, Chirapat & Lewmanomont, 2014	*Gp.mclachlanii* Buriyo, Bellorin & M.C.Oliveira in Bellorin et al. 2008	*Gp.nhatrangensis* Nhu Hau Le & Showe M.Lin 2006
Type locality	Tamarind Beach, Lembongan Island, Bali, Indonesia	Wu-Shih-Bi Harbor, Tou-Cheng Township, Taiwan	Enoura, Namazu city, Shizuoka Pref., Japan	Yinggehai, Hainan, Guangdong, China	Ao Len, Trat Peninsula, Thailand	Nungwi Maraní, Unguja Island, Zanzibar, Tanzania	Cua Be, Nha Trang, Southern Vietnam
Thallus length	up to 13 cm	up to 22 cm	up to 200 cm	up to 70 cm	up to 10 cm	>150 cm	up to 18 cm
Main axes	percurrent, terete, 640 μm in diam.	terete, 3 mm in diam., up to 8 main axes arising from a holdfast	more or less percurrent, cylindrical, compressed, up to 5 mm in diam.	percurrent or not, cylindrical, up to 3 mm in diam.	percurrent, cylindrical, 2–2.5 mm in diam.	percurrent or not, cylindrical throughout, 1–3 mm in diam.	cylindrical to terete, up to 2.2 mm in diam., up to 15 main axes arising from a holdfast
Indeterminate branches	sparsely irregular	5–7 densely clustered in the middle of axes, regenerating branches at the tip	alternate or irregular, long	long, up to four orders, easily broken, irregularly alternate, secund or furcate	very irregular to secund, sometimes inflated in middle, up to third orders	unbranched at the base, scattered, alternate to irregular, up to four orders	branched 1–2 orders from the base
Determinate branches	filiform, up to 5 mm long, scarce, irregular	absent	filiform, absent in young thalli, but often numerous, short	short, spinose, gradually tapered, non-constricted at the base	short, sometimes spine-like, frequently crowded	absent	numerous, racemose
Cortex	1–2 layers of small cells	up to 3 layers of ovoid cells	up to 3 layers of globular cells with dense cytoplasm, with the subcortex of 3–4 layers of elongated cells	2–3 layers of small, roundish cells	1–2 layers of cortical cells	2 layers of isodiametric to elongate cells, with the subcortex of 1–3 layers	3–4 layers of ovoid cells, 6–7 μm in diam., with the subcortex of 1–3 layers
Medulla	large, thin-walled cells	large thin-walled cells	5–7 layers of large, polygonal to spherical, vacuolated cells	large, parenchymatous cells	5–12 cell layers	Large globose cells, thin-walled, highly vacuolated	large, thin-walled, vacuolated cells
Cystocarp	hemispherical, scattered on main axes or branches	dome-shaped, broad-based, carposporangia in branched chains	slightly beaked, constricted at bases	prominently protruding or subconical, around 1,000 μm in diam., non-constricted at the base	dome-shaped, not constricted, 200 μm in diam.	prominent, not constricted at the base	prominent, not constricted at the base
Pericarp	9–14 cell layers	10–14 cell layers	6–8 cell layers	7–8 cell layers	10–12 cell layers	8–13 cell layers	11–17 cell layers
Spermantangia	not found	not found	scattered, continuous over branch surface	scattered, continuous over branch surface	continuous or discontinuous cluster	scattered, irregular pale patches	superficial
Tetrasporangia	scattered, cruciate, 20–34 × 19–24 μm in size	scattered, cruciate, 40–50 × 25–30 μm in size	scattered on surface, cruciate, 46–56 × 26–35 μm in size	scattered, cruciate to irregularly tetrahedral, 33–36 × 16–26 μm in size	cruciate, 28–35 µm in diam.	scattered, decussate to cruciate, 20–60 × 15–32 μm in size	cruciate, 20–30 × 10–20 μm in size
Distribution	Southern Indonesia	Taiwan, Japan	China, Japan, Korea	China, Malaysia, Philippines	Thailand	Tanzania	Vietnam
Reference	This study	Lin 2008; Yang and Kim 2015	Yamamoto 1978; Kim et al. 2008	Zhang and Xia 1988; Hurtado-Ponce and Liao 1998; Yang and Kim 2015	Abbott 1988; Muangmai et al. 2014	Bellorin et al. 2008	Le and Lin 2006

#### ﻿ Gracilariopsis grevogerungii

Taxon classificationPlantaeGracilarialesGracilariaceae

G.H.Boo & I.K.Hwang sp. nov.

68F9B310-9ED1-5C47-BAE5-E8AB15D0AE09

Figs 3 , 4

##### Description.

Thallus up to 13 cm tall, solitary, yellow-green to pale red in color. Main axes cylindrical throughout, about 640 μm in diameter, arising from a small disk-like holdfast (Fig. 3). Indeterminate branches often unbranched at the base, scattered, alternate to irregularly arising to 1–2 orders, slightly constricted at the base and tapering gradually toward apices. Determinate branches up to 4 mm long, irregularly alternate or scattered. Cortices composed of two to three layers of small isodiametric or anticlinally elongate cells (Fig. 4A, B), measuring 3.2–6.4 × 7.5–12.8 μm in size, with dense content, heavily pigmented, connected only with their parental cells by primary pit connections. Medulla composed of large globose cells, 114–163 μm in diameter in transverse sections, thick-walled and highly vacuolated, lacking pigments. Transition in cell size from cortex to medulla abrupt (Fig. 4C). Large basal cells of deciduous hairs frequently occurring near the surface. Cystocarps 492–805 μm in diameter and 689–884 μm in height, hemispherical, scattered on main axes or determinate branches, slightly constricted at the base. Cystocarps slightly constricted at the base, with up to three ostioles (Fig. 4D, E). Carposporangia initials formed in long chains and radially elongated (Fig. 4F, G). Two gonimoblasts formed in a single cystocarp (Fig. 4H). Mature cystocarps released carpospores through ostioles (Fig. 4I). Pericarps about 115 μm thick, formed by 9–14 cell layers. Tetrasporangia embedded in the cortex (Fig. 4J). Tetrasporangial initials formed from inner cortical cells (Fig. 4K). Tetrasporangia decussately or cruciately divided, ovoid, 20–34 × 19–24 μm in size (Fig. 4L, M).

##### Diagnosis.

Diagnosed by a combination of characters: simple terete axes with irregular long branches and scarce short branchlets, hemispherical cystocarps with up to 3 ostioles, 9–14 cell-layered pericarp, and DNA sequences (accession number: PV106182 for *rbc*L and PV106180 for COI-5P).

##### Type.

Indonesia • Bali, Nusa Lembongan Island, Tamarind Beach, 8°40'47.51"S, 115°26'10.20"E, 21 Jun. 2017, collected by Sung Min Boo without collection numbers (Holotype: PKNU00673!; Isotypes: PKNU00672!, PKNU00674!, and PKNU00676!; Paratype: PKNU00675!). Types are deposited in the herbarium of the Department of Marine Biology, Pukyong National University, Busan, Korea (PKNU).

##### Habitat and distribution.

*Gracilariopsisgrevogerungii* grows on intertidal to shallow subtidal sandy-muddy substrates. It is currently demonstrated in the type locality solely using DNA sequences, but its range is likely expanded to the surrounding waters with additional collections (see Discussion).

##### Etymology.

Species epithet is given in honor of Dr Grevo Soleman Gerung for his contributions to the knowledge of seaweed diversity in Indonesia.

## ﻿Discussion

*Gracilariopsisgrevogerungii* is the only described southern Indonesian species of *Gracilariopsis* investigated by both *rbc*L and COI-5P sequences, as well as morphology. The cystocarp anatomy revealed the chains of carposporangia and the lack of nutritive tubular cells, typical characters of *Gracilariopsis*. *Gracilariopsisgrevogerungii* was likely misidentified as *Gp.lemaneiformis* based on specimens collected in Flores Island and Tanah Djampea, Indonesia (Weber-van Bosse 1928). Its illustration of cystocarp having 10–17 cell layers in pericarp and lacking the nutritive tubular cells matches well with *Gp.grevogerungii*.

*Gracilariopsisgrevogerungii* has likely been misidentified as terete species of *Gracilaria*. For example, during the present study, *Gracilariaedulis* (S.G.Gmelin) P.C.Silva was collected in Benoa Bay, Bali, very close to Nasa Lembongan Island. It is a common species in Indonesia and also included in the export list of Indonesian hydrocolloid seaweeds (Meinita et al. 2021; Basyuni et al. 2024). However, COI-5P sequences from our collections of *G.edulis* (GHB, unpubl.) revealed its difference from *Gp.grevogerungii*. Again, *Gracilaria* is well segregated from *Gracilariopsis* by the presence of nutritive filaments in the cystocarp and by molecular data (Gurgel et al. 2003b; Bellorin et al. 2008).

*Gracilariopsis* sp. from Zamboanga city, Philippines, which lacks fine, determinate branchlets (Hurtado-Ponce and Liao 1998), is morphologically similar to *Gp.grevogerungii*. DNA sequences from the Philippine collection are necessary to confirm its identity. *Gracilariopsislemaneiformis* has still been reported in China (Wang et al. 2023), even though it has been confirmed by *rbc*L sequences that the Chinese specimens do not correspond to *Gp.lemaneiformis* (whose type locality is in Peru), but to *Gp.chorda* (whose type locality is in Japan). Additional sampling at sand-muddy coastal regions will likely extend the range of *Gp.grevogerungii* into other locations in Indonesia or surrounding waters.

Two species of *Gracilariopsis* were closely related to *Gp.grevogerungii* in both *rbc*L and COI-5P phylogenies. *Gracilariopsismclachlanii*, originally described for Tanzanian specimens, is recognized by large cylindrical form of thallus, 8–13 cell layers of pericarp, and cleavage of spermatangia from spermatangial mother cells through concavo-convex oblique septa (Bellorin et al. 2008). *Gracilariopsisheteroclada*, first described for Chinese specimens, is characterized by slender, filiform indeterminate branches that arise alternatively and are beset with fine, regularly-disposed determinate branchlets (Zhang and Xia 1988; Hurtado-Ponce and Liao 1998).

Several species of *Gracilariopsis*, including *Gp.heteroclada*, have been previously recognized in Southeast Asia (Pham 1969; Le and Lin 2006; Lin 2008; Muangmai et al. 2014). Most species except foliose *Gp.mageshimensis* Mas.Suzuki & R.Terada from Japan resemble *Gp.grevogerungii* in their habits with filiform thalli having irregular lateral branches from main axes (Table 2). Of these, *Gp.chiangii* Showe M.Lin, *Gp.heteroclada*, and *Gp.nhatrangensis* Nhu Hau Le & Showe M.Lin have been well delimited by DNA sequences and morphology (Le and Lin 2006; Lin 2008; Wang et al. 2023). *Gracilariopsischiangii* is characterized by small-sized thallus (15–22 cm in length) and 1–2(–3) orders of branches from the base to middle part of thallus (Lin 2008). *Gracilariopsisnhatrangensis* is characterized by 1–15 main branches (up to 18 cm tall), arising from a discoid holdfast and numerous, racemose branchlets on main axes (Le and Lin 2006).

*Gracilariopsisirregularis* from Thailand is distinguished by having short (up to 10 cm) and succulent axes with an irregular secondary branching pattern (Muangmai et al. 2014). However, *Gp.irregularis* requires reexamination by COI-5P and other molecular markers because of its homogeneity to *Gp.heteroclada* in *rbc*L. It is important to reexamine the holotypes or topotype materials of two Vietnamese species, *Gp.nganii* Pham and *Gp.phanthiens* Pham, which have not been recorded since the original publication (Le and Lin 2006; Pham 1969), to elucidate their relationships with *Gp.grevogerungii* and other *Gracilariopsis* species. Because most *Gracilariopsis* species have similar vegetative traits (Bellorin et al. 2008), it is difficult to discriminate *Gp.grevogerungii* from other cylindrical species of *Gracilariopsis* in Southeast Asia without DNA sequences.

## ﻿Conclusions

DNA sequence analyses were essential for the recognition of a new tropical species, *Gracilariopsisgrevogerungii*, from southern Indonesia. This study highlights the critical role of molecular data in elucidating the biodiversity of a morphologically simple group of marine red algae. *Gracilariopsisgrevogerungii*, along with *Gracilaria* species, is expected to be included in the export list of Indonesian hydrocolloid seaweeds. Our integrative taxonomy, combining molecular and morphological evidence, contributes to the clarification of commercial seaweed species and the cataloguing of red algal biodiversity. This study emphasizes the necessity for continued taxonomic and molecular investigations of the agar-producing genus *Gracilariopsis* in Indonesia and adjacent regions.

## Supplementary Material

XML Treatment for Gracilariopsis grevogerungii

## ﻿Additional information

### Use of AI

No use of AI was reported.

### Author ORCIDs

Ga Hun Boo https://orcid.org/0000-0002-3019-2678

Il Ki Hwang https://orcid.org/0009-0005-5120-0891

### Data availability

All of the data that support the findings of this study are available in the main text.
